# Validity of polymyalgia rheumatica diagnoses and classification criteria in primary health care

**DOI:** 10.1093/rap/rkz033

**Published:** 2019-08-27

**Authors:** Charlotta Fors, Ulf Bergström, Minna Willim, Eva Pilman, Carl Turesson

**Affiliations:** 1 Department of Rheumatology, Skåne University Hospital; 2 Rheumatology, Department of Clinical Sciences, Malmö, Lund University, Malmö; 3 Vårdcentralen Landborgen, Helsingborg, Sweden

**Keywords:** polymyalgia rheumatica, PMR, classification criteria, primary health care, primary care

## Abstract

**Objectives:**

PMR is an inflammatory disease with prominent morning stiffness and muscular tenderness, usually diagnosed in primary health care (PHC). The objectives were to examine the validity of PMR diagnoses in PHC and to validate the use of classification criteria for PMR.

**Methods:**

Medical records for patients with a registered PMR diagnosis at two PHC facilities were reviewed. Patients were classified according to several sets of criteria. An independent review, with assessment of the PMR diagnosis, was performed by an experienced rheumatologist.

**Results:**

Of 188 patients, the PMR diagnosis was in agreement with the independent review in 60% overall, in 84% of those fulfilling a modified version of the ACR/EULAR classification criteria and in 52% of those who did not. The corresponding proportions for the Bird criteria were 66 and 31%, and for the Healey criteria 74 and 42%. In 74% of the medical records, documentation on morning stiffness was missing. Rheumatoid factor was tested in 22% and anti-CCP antibodies in 15%.

**Conclusion:**

In this study of patients with PMR diagnosed in PHC, the diagnosis was supported by the independent review in 60% of the patients. Documentation on morning stiffness and testing for autoantibodies were limited. A modified version of the ACR/EULAR criteria can be used to identify patients with a valid PMR diagnosis in retrospective surveys but does not capture all PMR patients. The modified ACR/EULAR criteria may be more stringent than some of the older criteria sets.

**Key messages**

- The PMR diagnosis was supported in 60% of the patients initially diagnosed in primary health care.
- The ACR/EULAR criteria for PMR appear to be more stringent than some older criteria set.

## Introduction

PMR is an inflammatory disease characterized by subacute to acute onset of pain in the neck, shoulders, upper arms, pelvis and thighs, with prominent morning stiffness and tenderness [1, 2]. PMR almost exclusively affects individuals >50 years of age [3, 4], and it is two to three times more common in women [4]. It usually presents with laboratory signs of inflammation, including elevated ESR and CRP [1]. Other manifestations of systemic inflammation, such as low-grade fever, weight loss and fatigue, are present in ≤40% of PMR patients [4]. Studies using different imaging techniques have shown joint involvement in patients with PMR [5–7]. An early phase of elderly-onset RA can sometimes mimic the clinical characteristics of PMR. Anti-CCP antibodies in serum, with high specificity for RA, can be useful in differientiating between PMR and elderly-onset RA [8, 9]. Other medical conditions that need to be excluded are crystalline arthropathies, degenerative joint disease, frozen shoulder, malignant diseases, parkinsonism, thyroid diseases and drug-induced myopathies, among others [1]. Genetic and environmental factors, including infections, have been implicated in the aetiology of PMR [1, 4, 10]. Furthermore, depression has been suggested to be more common in patients with PMR than in the general population of older adults, regardless of current glucocorticoid dosage [11].

The diagnosis of PMR is based on the characteristic symptoms together with elevated acute phase reactants, adequate response to glucocorticoid treatment and the absence of other conditions with similar presentation [10]. There are five sets of criteria available to classify PMR [1, 12–16], the most recent from 2012 being the result of a collaboration between ACR and EULAR [16] (Table 1). The criteria from ACR/EULAR include a variant in which ultrasonography is used to detect local signs of inflammation in shoulders and hips [16]. Two prospective studies from rheumatology care specialist settings have compared sensitivity and specificity for the different sets of classification criteria and their ability to discriminate PMR from other conditions that could mimic the diagnosis at presentation. The findings were divergent regarding which criteria had the overall highest discriminating ability, although both suggested that US could increase the specificity for the 2012 ACR/EULAR criteria [17, 18].

**Table 1:** Different sets of criteria for the diagnosis and classification of PMR

	Bird criteria, 1979	Jones and Hazleman criteria, 1981	Chuang and Hunder criteria, 1982	Healey criteria, 1984	ACR/EULAR criteria, 2012
Disease onset	Onset of illness within 2 weeks	Disease duration >2 months	Bilateral aching and stiffness ≥ 1 month in two of the following: neck/torso, shoulders/upper arms, hips/thighs	Persistent pain ≥1 month in two of the following: neck, shoulders, pelvic girdle	
Morning stiffness	Morning stiffness >1 h	Morning stiffness >1 h		Morning stiffness >1 h	Morning stiffness >45 min (two points)
Shoulder/pelvic/hip pain	Bilateral shoulder pain with/without stiffness	Shoulder or pelvic girdle pain without muscle weakness			Hip pain/restricted range of motion (one point)
Upper arm tenderness	Bilateral upper arm tenderness				
Exclusions by signs/symptoms/laboratory values		Absence of objective signs of muscle disease			Absence of other joint involvement (one point)
					Absence of RF or anti-CCP antibodies (two points)
Age at onset	Age ≥ 65 years		Age ≥ 50 years		
ESR/CRP	Initial ESR ≥40mm/h	ESR >30 mm/h or CRP >6 mg/l	ESR >40 mm/h	ESR >40 mm/h	
Absence of other rheumatic diseases		Absence of RA	Exclusion of other diagnosis, with the exception of GCA	Absence of other joint or musculoskeletal diseases	
Response to glucocorticoids		Fast and dramatic response to systemic glucocorticoids		Rapid response to prednisolone (≤20 mg/day)	
Signs on ultrasonography					If ultrasonography available, ≥ one shoulder with subdeltoid bursitis, biceps tenosynovitis or glenohumeral synovitis (posterior/axillary); and ≥ hip with synovitis/trochanteric bursitis (one point)
					If ultrasonography available, both shoulders with subdeltoid bursitis, biceps tenosynovitis or glenohumeral synovitis (one point)
Depression/weight loss	Depression/weight loss				
Requirements for diagnosis	Diagnosis of probable PMR requires at least three of the above criteria	All the above criteria required for diagnosis of PMR	All the above criteria required for diagnosis of PMR	Diagnosis of PMR requires the age of ≥50 years and the fulfilment of at least three criteria	Diagnosis with PMR requires the age of ≥50 years, bilateral shoulder aching, abnormal CRP/ESR levels and at least four points (without ultrasonography)/at least five points (with ultrasonography)

PMR is most often diagnosed in primary health care (PHC). This underlines the need for studying the utility and validity of the classification criteria in such a setting. The objectives of the study were to examine the validity of PMR diagnoses in PHC and to validate the use of classification criteria for PMR in a retrospective survey of a PHC cohort.

## Methods

### Source population

Patients with a registered diagnosis of PMR [International Classification of Diseases, 10th edition (ICD-10) M353, PMR or M315, GCA with PMR) between 2000 and 2013 at the PHC facilities Stattena and Tågaborg in the city of Helsingborg (northwestern Skåne, Sweden) were identified using the patient administrative system. Registration of diagnostic codes in public health care is mandatory in Sweden. The proportion of physician visits in public primary health care in the region with registered diagnostic codes was ∼90% in 2004–2010 [19] and has remained at ≥ 80% until 2017.

### Data collection

Based on the subsequent review of medical records, patients diagnosed with PMR before 2000 or at another health=care facility without the possibility of tracking information were excluded. All the medical records from primary care were systematically reviewed, and additional information was collected from the regional, hospital-based computerized medical record system (Melior/Sieview) when indicated. A form was completed for the patients meeting the inclusion criteria of being diagnosed with PMR between 2000 and 2013 and for whom appropriate information was available. A subset of the patients (*n* = 48) was reviewed by two investigators (C.F. and U.B.), and the proportions with agreement on fulfilment of classification criteria were calculated. The reference method for verification of the PMR diagnosis was an independent review, with assessment of the long-term disease course and differential diagnoses, by an experienced rheumatologist with access to all electronic records.

### Classification

Components for the PMR classification criteria by ACR/EULAR [16], Bird *et al.* [12] and Healey [15] were constructed. Variables for the criteria by Jones & Hazleman [13] and Chuang *et al.* [14] were not constructed, because the patient material contained insufficient information to assess whether the criteria could be fulfilled. Specifically, data were missing about aching and stiffness in specific areas and regarding the duration of symptoms overall. According to different classification criteria, morning stiffness should have a duration of 45 min [16] and 60 min [12, 13, 15], respectively. Given that none of the medical records reviewed had information about the duration of morning stiffness, it was considered relevant if present.

### Statistics

Descriptive statistics were obtained separately for all included patients and for those who had a PMR diagnosis verified by an expert in rheumatology in an independent review or who fulfilled the classification criteria by ACR/EULAR [16], Bird *et al.* [12] or Healey [15]. For the present study, a modified version of the ACR/EULAR criteria [16] was constructed, requiring two points for a PMR diagnosis if RF or anti-CCP was unavailable. If test results on RF or anti-CCP were available, four points were required, as in the original criteria. Proportions fulfilling of each of these sets of criteria among those with a diagnosis that was supported by the independent review were calculated.

### Ethics

The study was approved by the regional ethical review board in Lund, Sweden (ref. 2014/760; 27 November 2014).

## Results

### Patients

For the PMR cohort, 305 patient records were subjected to a structured review. A total of 117 were excluded, of which 66 had been diagnosed with PMR before the year 2000 and 24 had received the diagnosis at another health-care facility. Furthermore, 16 records were considered to have an insufficient amount of information available for completing the form, and 11 patients had been registered incorrectly as PMR without having the diagnosis or the symptoms of PMR or GCA and were thus excluded. Therefore, 188 patients (75% females) with a diagnosis of PMR at the two PHCs between 2000 and 2013 were included (Table 2).

**Table 2:** Medical history and clinical findings in patients with PMR by agreement on diagnosis or fulfilment of classification criteria

	All	**Supported** ^a^ **PMR**	ACR/EULAR fulfilled	Bird fulfilled	Healey fulfilled
*N*	188	113	49	145	93
Female sex, % (*n*)	75 (140)	68 (77)	70 (34)	73 (106)	70 (65)
Age^b^, mean (s.d.), years	75.6 (9.9)	75.3 (8.8)	74.4 (7.8)	76.6 (8.7)	74.5 (9.8)
Ongoing glucocorticoid treatment at review, % (*n*)	50 (94)	51 (58)	43 (21)	52 (76)	38 (35)
Documented depression, % (*n*)	37 (69)	38 (43)	33 (16)	41 (59)	38 (35)
Documented significant weight loss, % (*n*)	28 (53)	27 (31)	18 (9)	33 (48)	30 (28)
Elevated ESR or CRP^b^, % (*n*)	90 (169)	98 (111)	100 (49)	92 (133)	91 (85)
ESR^b^, mean (s.d.), mm in first hour	62 (26)	60 (26)	60 (29)	63 (27)	61 (21)
CRP^b^, median (IQR), mg/l	58 (44–92)	57 (34–87)	57 (32–92)	58 (35–93)	57 (34–80)
RF positive, % (*n*/*N*)	2 (4/42)	0 (0/25)	0 (0/24)	2 (3/35)	0 (0/24)
Anti-CCP positive, % (*n*/*N*)	0 (0/29)	0 (0/16)	0 (0/17)	0 (0/21)	0 (0/16)
Rapid response to cortisone, valid % (*n*)	44 (82)	52 (59)	66 (55)	45 (65)	70 (65)
Symptomatic response to cortisone, valid % (*n*)	89 (167)	97 (109)	98 (45)	90 (130)	93 (86)

Values are expressed as the total percentage unless otherwise indicated.

Missing data (all): elevated ESR/CRP at onset, *n* = 3; ESR at inclusion, *n* = 31; CRP at inclusion, *n* = 27; cortisone rapid response, *n* = 31; cortisone symptomatic response, *n* = 1.

a By an expert in rheumatology in an independent review.

b At onset of PMR.

IQR = interquartile range.

### Patient characteristics

The mean age at PMR diagnosis was 75.6 years (s.d.: 9.9 years). Depression and weight loss were documented in 37 and 28% of patients, respectively. RF and anti-CCP antibodies were analysed in <25% of the patients (Table 2). The mean age was slightly lower among those tested for RF (70.5 years) and among those tested for anti-CCP (69.3 years) compared with all patients in the cohort (75.0 years). There was also a slightly lower proportion of female patients among those tested for RF (69%), but not among those tested for anti-CCP (76%) compared with the cohort overall (75%). Only four patients were positive for RF; none was positive for anti-CCP. Morning stiffness was reported by 20% of the patients, with 73% of the medical records missing information on this symptom (Table 2).

### Classification

A total of 26% fulfilled the ACR/EULAR criteria. The corresponding proportions for the Bird criteria and the Healey criteria were 77 and 49%, respectively. The age and sex distributions were similar in those who fulfilled the different sets of PMR classification criteria examined (Table 2). Of those fulfilling the ACR/EULAR, Bird and Healey classification criteria, 66, 45 and 70%, respectively, responded rapidly to CSs.

Among the 48 patients reviewed by two investigators, agreement on fulfilment of the ACR criteria was 81%, whereas the corresponding proportions for the Bird criteria and the Healey criteria were 73 and 70%, respectively.

### Validity

**Fig. 1:**
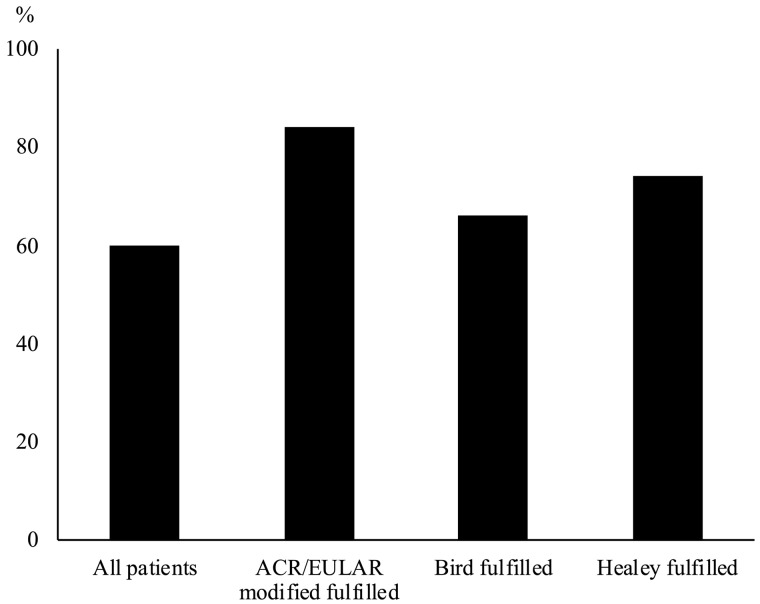
Supported diagnosis by subset of PMR Proportion of patients with a diagnosis of PMR that was in agreement with an independent review by an experienced rheumatologist, among all patients and by fulfilment of criteria.

Of the 188 patients included, the PMR diagnosis was in agreement with the independent review in 113 cases (60%). The corresponding proportions for those classified as PMR were 84% for the ACR/EULAR criteria, 74% for the Bird criteria, and 66% for the Healey criteria (Fig. 1). The proportions with a supported diagnosis were higher among those fulfilling the ACR/EULAR criteria (84 *vs* 52%), the Bird criteria (66 *vs* 31%) and the Healey criteria (74 *vs* 42%) compared with those not fulfilling each set of criteria. Among those with a PMR diagnosis that was not supported by the independent review, the diagnosis was subsequently changed at the PHC in 17 of 75 cases (23%).

Compared with the overall cohort, those with a supported PMR diagnosis had a similar mean age at onset, but a lower proportion of females (68 *vs* 75%). No apparent difference was found between the subsets in symptom duration at the onset of PMR (Table 3). The vast majority of patients had an elevated ESR or CRP, occurring in 90% of the total group and in 98% of those with supported PMR. Symptomatic response to CSs was reported in 89% of all patients, in 97% of those with a supported PMR diagnosis and in 98% of those fulfilling the ACR/EULAR criteria. A rapid response to CSs, defined as within 7 days of prednisolone treatment at <21 mg/day, was seen in 44% of all patients and in 52% of those with supported PMR.

**Table 3:** Symptoms at presentation in patients with PMR by agreement on diagnosis or fulfilment of classification criteria

	All	**Supported** ^a^ **PMR**	ACR/EULAR fulfilled	Bird fulfilled	Healey fulfilled
*N*	188	113	49	145	93
Onset of illness <2 weeks, % (*n*)	27 (51)	30 (34)	33 (16)	34 (49)	18 (17)
Duration of symptoms at PMR diagnosis, median (IQR), days	21 (14–41)	21 (14–45)	21 (12–42)	21 (13.5–36.3)	30 (14–60)
Unilateral shoulder pain, % (*n*)	11 (20)	12 (14)	0 (0)	7 (10)	11 (10)
Bilateral neck pain, % (*n*)	29 (55)	32 (36)	35 (17)	30 (44)	31 (29)
Unilateral neck pain, % (*n*)	1 (2)	1 (1)	0 (0)	1 (2)	0 (0)
Bilateral upper arm tenderness, % (*n*)	37 (70)	47 (53)	51 (25)	43 (63)	45 (42)
Pelvic girdle muscle pain, % (*n*)	64 (121)	79 (89)	76 (37)	65 (94)	82 (76)
Morning stiffness, % (*n*)	20 (38)	27 (30)	51 (25)	24 (35)	38 (35)
Morning stiffness neck/torso, % (*n*)	2 (4)	2 (2)	4 (2)	3 (4)	4 (4)
Morning stiffness shoulder/upper arms, % (*n*)	6 (11)	8 (9)	16 (8)	7 (10)	11 (10)
Morning stiffness hips, % (*n*)	4 (7)	6 (7)	8 (4)	4 (6)	7 (6)
Hip pain/decreased movement, % (*n*)	37 (70)	48 (54)	51 (25)	36 (52)	42 (39)

Missing data (all): symptom duration, *n* = 55; morning stiffness, *n* = 138.

a By an expert in rheumatology in an independent review.

IQR=interquratile range.

## Discussion

Sixty per cent of the patients had a PMR diagnosis that was in agreement with the review by the rheumatologist. Among the sets of criteria for PMR, the proportion with a supported diagnosis was the greatest among those fulfilling the ACR/EULAR classification criteria (84%). In this cohort, PMR was mainly diagnosed in patients who were in their 70s. As expected, the majority were female, although there was a higher proportion of men in those with verified PMR compared with patients who had a PMR diagnosis overall. The findings of a mean age of 76 years at PMR onset and a proportion of 75% females are compatible with previous studies. The vast majority of patients diagnosed with PMR had elevated ESR and/or CRP and responded to glucocorticoid therapy.

Morning stiffness is often regarded as a key symptom of PMR. One study has reported a significant diurnal variation in some cytokines, CRP and cortisol, with higher levels in 10 patients with PMR compared with a control group. This provides a possible explanatory model for the morning stiffness in PMR [20]. From the cohort of 188 patients identified in PHC facilities and finally included in the study, only 20% fulfilled the criterion of morning stiffness. No more than 6% had denied the presence of morning stiffness, and thus a total of 138 (74%) were labelled as missing. Morning stiffness is a variable included in four of the different sets of classification criteria [12, 13, 15, 16], of which the set from Jones & Hazleman [13] requires complete fulfilment for a patient to be diagnosed with PMR. Nevertheless, there was little information available on the presence of morning stiffness in the 188 medical records that were finally included. This might be attributable, in part, to divergent opinions on the importance of morning stiffness, as reflected by a survey of international experts, where both rheumatologists and non-rheumatologists were asked to identify and rate potential classification criteria for PMR. With regard to morning stiffness the level of agreement was low, with only 57% of the non-rheumatologists believing it to be useful compared with 77% of the rheumatologists. Testing for anti-CCP antibodies seemed to be considered less important by non-rheumatologists (24%) than by rheumatologists (40%) for diagnosis of PMR [21].

A higher frequency of patients diagnosed with PMR might fulfil the classification criteria if the question about morning stiffness was asked more often and the response routinely documented. RF and anti-CCP antibodies were tested in a minority of the patients. These were, on average, slightly younger at diagnosis, possibly suggesting that RA might be considered a more relevant differential diagnosis in younger patients.

Furthermore, 29% of the medical records were missing information about symptom duration at onset, which is part of several of the classification criteria [12–15]. These findings suggest that there is a need to use structured guidelines for the diagnosis and follow-up of PMR in a primary health-care setting, because using a structured decision guidance document for data collection on PMR in clinical practice might improve quality of care.

Limitations of the study include the high frequency of missing data regarding the duration of symptoms and presence of morning stiffness in the PMR cohort. The use of a complete review of the records as the reference method is also a limitation. The study does not judge the accuracy of the diagnoses in PHC, but the PHC records.

Strengths of the study are related to the use of community-based cohorts of patients diagnosed in clinical practice. Selection bias is not a major issue, and the results are likely to be representative for patients with PMR in southern Sweden.

## Conclusion

The demographics of the cohort were consistent with previous studies of PMR. Of the 188 patient records finally included in the study, the PMR diagnosis could be verified by an experienced rheumatologist in 60% of them. This, together with the limited documentation on morning stiffness, underlines the heterogeneity of PMR patients and related diagnostic procedures in PHC. The findings suggest that PHC physicians might consider information about morning stiffness and autoantibodies less useful compared with the views of rheumatologists. The proportion with a supported diagnosis was greatest among the patients fulfilling the ACR/EULAR criteria, followed by the patients fulfilling the Bird criteria and the Healey criteria.
